# The inhibitory effect of pyrroloquinoline quinone on the amyloid formation and cytotoxicity of truncated alpha-synuclein

**DOI:** 10.1186/1750-1326-5-20

**Published:** 2010-05-20

**Authors:** Jihoon Kim, Ryuichi Harada, Masaki Kobayashi, Natsuki Kobayashi, Koji Sode

**Affiliations:** 1 Department of Biotechnology, Graduate School of Engineering, Tokyo University of Agriculture & Technology, 2-24-16 Naka-cho, Koganei-shi, Tokyo 184-8588, Japan

## Abstract

**Background:**

Parkinson's disease (PD) involves the selective damage of dopaminergic neuron cells resulting from the accumulation and fibril formation of alpha-synuclein. Recently, it has been shown that not only full-length alpha-synuclein, but also C-terminal truncated forms exist in the normal brain, as well as Lewy bodies, which are cytoplasmic inclusions in PD. It is known that truncated alpha-synuclein has a much higher ability to aggregate and fibrillate than full-length alpha-synuclein. Since the fibrils and precursor oligomers of alpha-synuclein are cytotoxic to the neuron, inhibitors that prevent the formation of oligomers and/or fibrils might open the way to a novel therapeutic approach to PD. However, no inhibitor for truncated alpha-synuclein has been reported yet.

**Results:**

In this study, we first characterized the aggregation and cytotoxicity of C-truncated alpha-synuclein119 and alpha-synuclein133 which have been found in both the normal and the pathogenic brain. Alpha-synuclein119 aggregated more rapidly and enhanced significantly the fibril formation of alpha-synuclein. Although both of alpha-synuclein119 and alpha-synuclein133 showed a high cytotoxicity, alpha-synuclein133 showed a similar aggregation with full-length alpha-synuclein and no acceleration effect. We showed that PQQ dramatically inhibits the fibril formation of C-terminal truncated alpha-synuclein110119, and 133 as well as the mixtures of full-length alpha-synuclein with these truncated variants. Moreover, PQQ decreases the cytotoxicity of truncated alpha-synuclein.

**Conclusions:**

Our results demonstrate that PQQ inhibits the amyloid fibril formation and cytotoxicity of the C-truncated alpha-synuclein variants. We believe that PQQ is a strong candidate for a reagent compound in the treatment of PD.

## Background

Parkinson' disease (PD) is a common neurodegenerative movement disorder affecting approximately 1% of the population above 65 years of age; it is a progressive neurodegenerative disease which develops slowly. The PD is caused by the specific and progressive degeneration of dopaminergic neurons in the substantia nigra. The neurodegeneration is accompanied by the presence of cytoplasmic inclusions, termed Lewy bodies (LBs). These inclusions are the hallmark pathological feature of PD [1-4]. The hypothesis according to which α-synuclein (α-Syn) plays a causative role in PD pathogenesis is strongly supported, since the major fibrillar protein component of LBs in both sporadic and familial PD is α-Syn, and three different α-Syn missense mutations (A30P, A53T and E46K) and the duplication and triplication of loci cause autosomal-dominant PD [5-9]. Several *in-vitro *studies have suggested that α-Syn's propensity to oligomerize and form fibrils may play a crucial role in its toxicity [10,12]. Current treatments are only symptomatic and do not stop or delay the progressive loss of neurons; there is as yet no preventative therapy available for PD.

α-Synuclein (140 aa) is a natively unfolded protein that is enriched in the presynaptic terminal of the neurons in the brain. The primary sequence of α-Syn is subdivided into three regions: the amphiphatic N-terminal region (residues 1-60), the highly hydrophobic central region called "NAC" (61-95), and the acidic C-terminal region (96-140) [13]. The C-terminal region is important for the high thermostability of α-Syn and for the chaperon activity [14]. Moreover, the C-terminal region regulates the amyloid aggregation and fibril formation of α-Syn. It is known from patients suffering from α-synucleinopathy that the C-terminal-truncated forms of α-Syn consist in LBs [15-18]. Several *in vitro *studies have shown the propensity of α-Syn to aggregate into amyloid fibrils, a process that is accelerated by the truncation of its C-terminal. In addition, when full-length α-Syn is mixed with C-terminal-truncated variants, the truncated α-Syn accelerate the aggregation of the full-length protein [17,19-21]. Recently, truncated α-Syn has been identified in the normal brain [18]. This indicates that the truncation of the C-terminal of α-Syn has some relevance to the pathogenesis of PD. Although many truncated variants have been characterized, some of the truncated α-Syn variants which exist in both the normal and the pathogenic brain, such as α-Syn119 and α-Syn133, have not yet been investigated through *in vitro *aggregation and fibril formation studies.

Neurodegenerative diseases, such as Parkinson' disease, Alzheimer's disease and prion disease, share the feature that the causative proteins change their conformation from the natural to the β-strand-rich conformation, acquiring oligomeric status, and subsequently forming supramolecular assemblies and amyloids. Considering that the formation of amyloid fibrils as well as their precursor oligomers is cytotoxic, agents that prevent the formation of the oligomers and/or fibrils might open the way to a novel therapeutic approach to these neurodegenerative diseases [22]. Therefore, considerable effort has been made to discover a molecule which prevents the amyloid formation of the causative proteins in these diseases, i.e., α-Syn [11,23], amyloid β [24,25] and prion protein [26], respectively.

**Figure 1:**
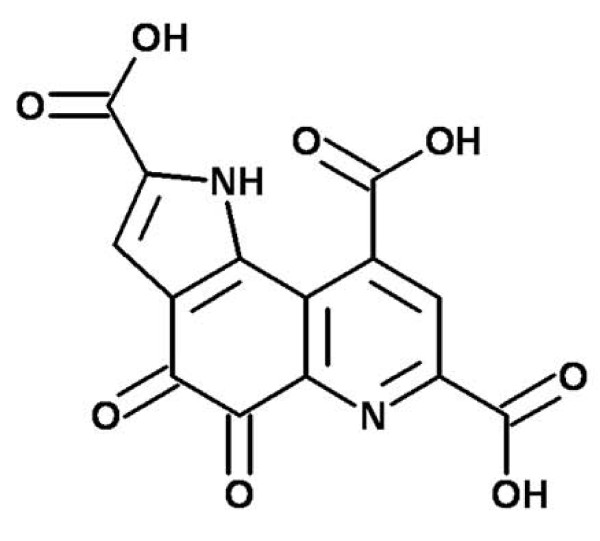
**Structure of pyrroloquinoline quinone**.

Previously, we studied the amyloid fibril formation mechanism of α-Syn and the development of a strategy for its prevention [27]. Also, we have reported that the antioxidant pyrroloquinoline quinone (PQQ) (Fig. 1), which is a noncovalently bound bacterial cofactor in the oxidative metabolism of alcohols, prevents the amyloid fibril formation and aggregation of full-length α-Syn *in vitro *in a PQQ-concentration-dependent manner [28]. PQQ forms a conjugate with WT α-Syn, and this PQQ-conjugated α-Syn is also able to prevent α-Syn amyloid fibril formation. The fact that PQQ shows anti-fibril-forming activity for α-Syn and other amyloid proteins and has never been reported, nor has a molecule which is able to prevent the amyloid formation of truncated α-Syn ever been described; this encouraged us to further investigate the effects of PQQ on the fibrillization and cytotoxicity of C-terminal-truncated α-Syn.

In this study, we first characterized the aggregation and fibrillation of truncated α-Syn119 and 133 as well as mixtures of full-length α-Syn with these truncated variants. We were able to demonstrate that PQQ dramatically inhibits the fibril formation of C-terminal-truncated α-Syn110, α-Syn119, and α-Syn133. Moreover, PQQ decreased the cytotoxicity of the truncated variants. Our results suggest PQQ has a strong potential as a relevant leading compound in the design of new amyloid inhibitors.

## Results

### Effect of truncation on the aggregation and fibril formation of α-synuclein

**Figure 2:**
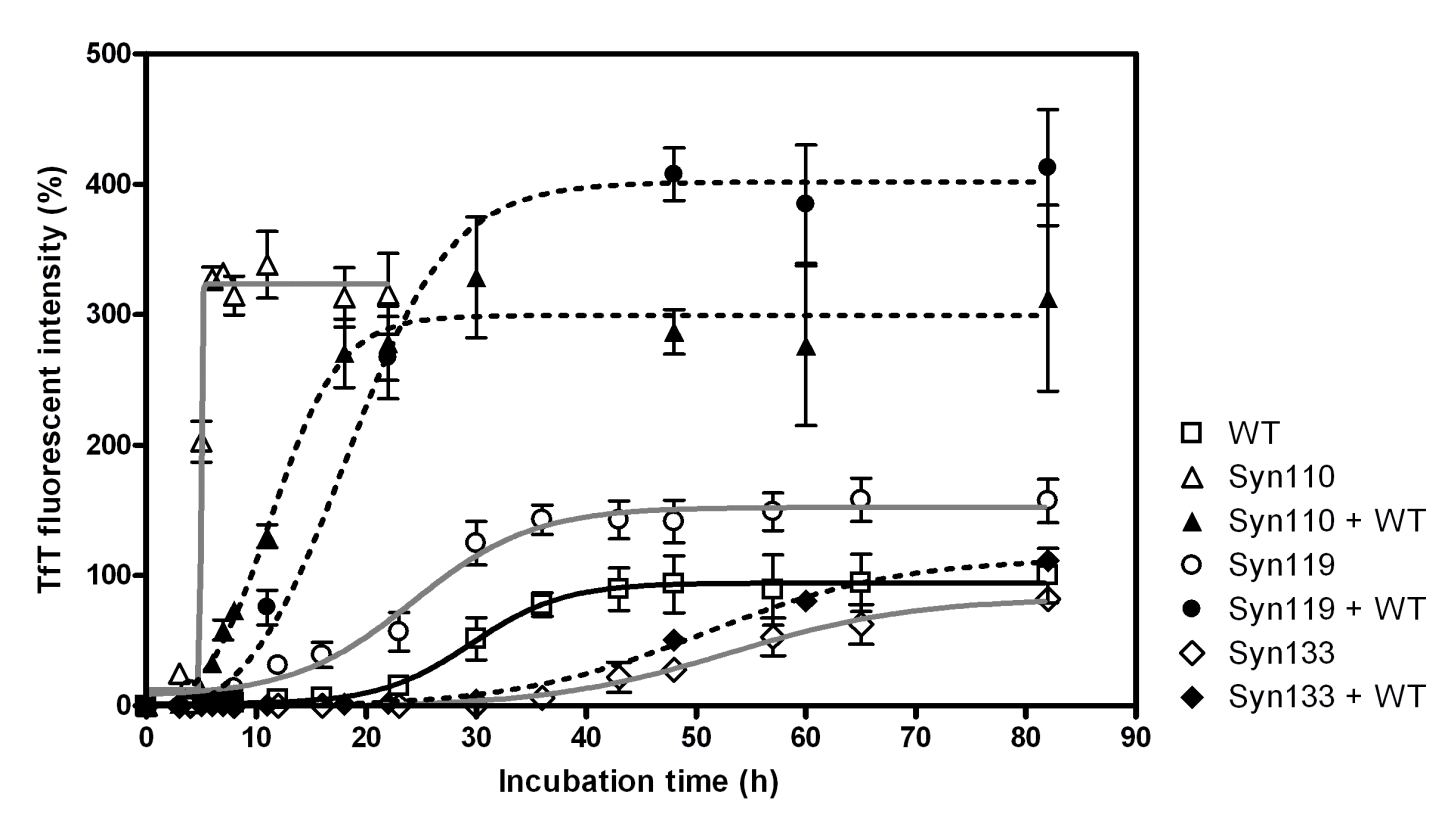
**Fibril formation of C-terminal-truncated variants and the mixture with full-length α-synuclein**. The time course of fibril formation was obtained from the TfT fluorescence assay analysis. Seventy μM protein was incubated in triplicate in PBS buffer with 0.02% NaN_3_, pH 7.4. Full-length α-Syn (white squares), α-Syn110 (white triangles), α-Syn119 (white circles), α-Syn133 (white diamonds), and 35 μM full-length α-Syn + either 35 μM α-Syn110 (black triangles) or 35 μM α-Syn19 (black circles) or 35 μM α-Syn133 (black diamonds).

**Figure 3:**
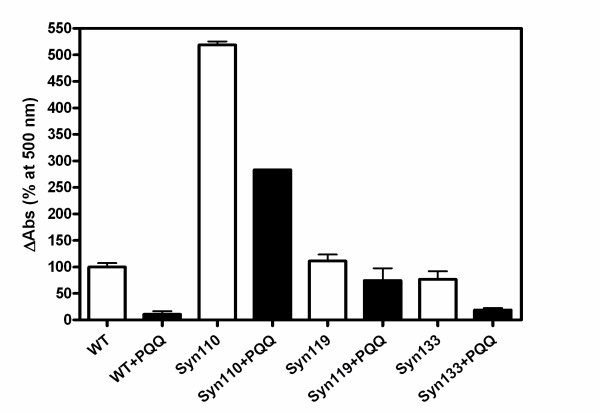
**Aggregation of C-terminal truncated α-synuclein**. Full-length α-Syn (70 μM) and truncated variants (70 μM) were incubated in PBS buffer, pH 7.4 with stirring at 37°C in the absence (white bar) or presence of 280 μM PQQ (black bar). Usually, the formation of total aggregations is monitored at 330 nm. However, PQQ has a typical absorbance at around 340 nm; so, the light scattering was monitored at 500 nm. n = 3 and error bar = standard deviation.

The binding of TfT to protein fibrils is accompanied by a characteristic increase in fluorescence intensity in the vicinity of 482 nm. (Fig. 2) compares the fibril formation patterns of full-length and truncated α-Syn110, α-Syn119, and α-Syn133, as monitored by TfT fluorescence. In agreement with earlier studies, the truncated α-Syn110 showed a much faster rate of fibril formation than full-length α-Syn. Also the fibril formation of truncated α-Syn119 was faster than that of full-length α-Syn. Meanwhile, the fibril formation of truncated α-Syn133 was slower than that of full-length α-Syn. The maximal values of fluorescence intensity in truncated α-Syn110, α-Syn119, and α-Syn133 were 3, 1.5 and 0.6-fold of full-length α-Syn and the lag time of truncated α-Syn was 4.8, 14.3 and 36.1 h respectively (full-length α-Syn, 21.4 h). The light scattering from protein solutions reflects the quantity of aggregates; i.e., this contained large soluble oligomers, amorphous aggregates and amyloid fibrils. The light scattering study showed that the aggregation ability of truncated α-Syn110 was higher than that of full-length α-Syn, but that the aggregation ability of truncated α-Syn119 and α-Syn133 was at same level as that of the full-length protein (Fig. 3).

### Truncated α-synuclein enhanced the fibril formation of α-synucleins

These truncated variants co-exist with full-length α-Syn in both the normal and the pathogenic brain. To investigate the effect of truncated α-Syn on the fibril formation of α-Syn, the fibrillation of a mixture of truncated and full-length α-Syn was monitored by TfT fluorescence and light scattering analysis. When truncated α-Syn110 and α-Syn119 coexisted with full-length α-Syn at a ratio of 1:1, the fibrillation rate increased and the lag time for fibrillation was reduced significantly, compared with those of full-length α-Syn ((Fig. 2) and Table 1).

**Table 1:** The kinetic analysis of α-Syn variants in this study.

Lag-time (h)	Full-length α-Syn	α-Syn110	α-Syn119	α-Syn133
**α-Syn (70 μM)**	21.4	4.8	14.3	36.1
**Truncated α-Syn (35 μM) +** **Full-length α-Syn (35 μM)**	-	5.0	8.8	31.4
**α-Syn (70 μM) +** **PQQ (280 μM)**	30.5	8.6	23.5	47.7

The lag time analysis of fibril formation was calculated from fitting-curve of TfT fluorescence was provided by PRISM (GraphPad Software)

Also, we performed the experiments to show the effect of truncated α-Syn with full length one. In order to show that the presence of truncated α-Syn enhances the fibril formation of α-Syn, various samples with different ratio of full length and α-Syn119 were prepared, fixing the final molar ratio at 70 μM α-Syn. If the presence of truncated α-Syn enhances the fibril formation of α-Syns, the amount of fibril should be higher than the sum of the amount of fibril formed with each α-Syn sample with each concentration. We used the amount of amyloid after 80 hours of incubation of the sample with 70 μM full length α-Syn alone as the control (100%). While the sample containing 35 μM of α-Syn119 alone resulted 120% of amyloid, the sample with 35 μM full length and 35 μM of α-Syn119 (total 70 μM) resulted 450% compared with the sample containing 70 μM of full length α-Syn.

**Figure 4:**
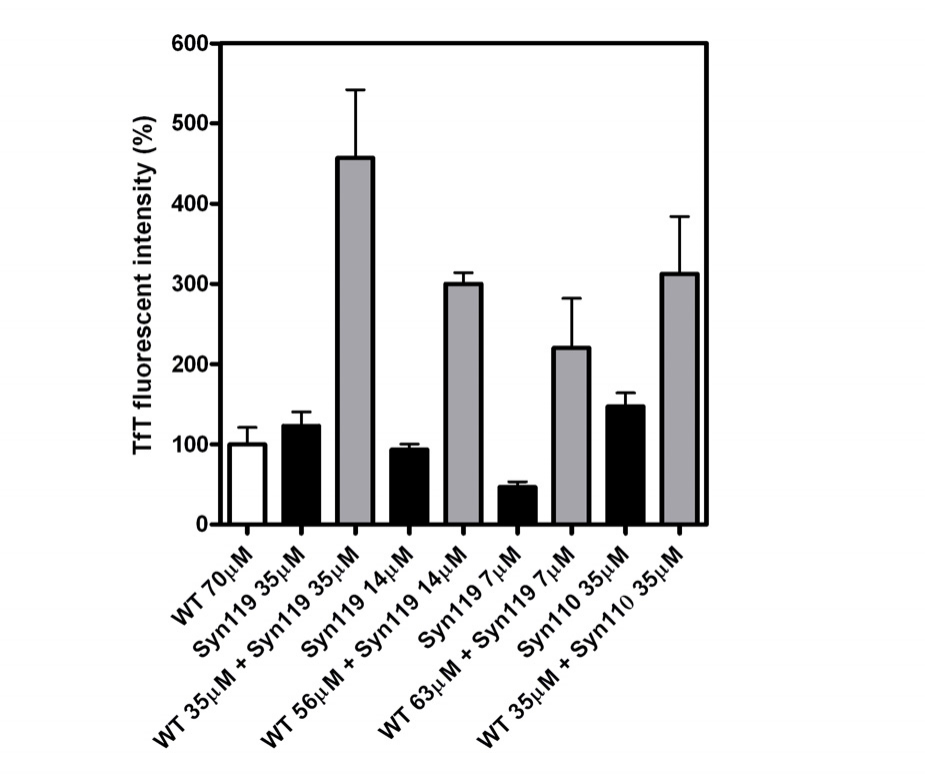
**Impact of C-terminal-truncated α-synuclein on the amyloid formation of full length of α-synuclein**. It showed the amount of amyloid after 80 hours of incubation of the sample with 70 μM full length α-Syn alone as the control (100%, white bar). 35, 14, and 7 μM of α-Syn119 and 35 μM of α-Syn110 (black bar) and the mixture protein (35 μM full-length α-Syn + 35 μM α-Syn119, 56 μM full-length α-Syn + 14 μM α-Syn119, 63 μM full-length α-Syn + 7 μM α-Syn119 and 35 μM full-length α-Syn + 35 μM α-Syn110, gray bar) was incubated in triplicate in PBS buffer with 0.02% NaN_3_, pH 7.4. The fibril formation was obtained from the TfT fluorescence assay analysis.

The observed amyloid formation was not the simple sum of the expected amount of amyloid forming from each α-Syn. The experiments were carried out with different mixing ratio, and similarly, the amount of fibril estimated from TfT fluorescence was not the simple sum and higher than those from the each α-Syn samples (Fig. 4). It is obvious that co-existence of full length α-Syn and truncated synuclein enhanced the total fibril formation of α-Syns. The presence of α-Syn110 also enhanced the total amyloid formation of α-Syn, but the effect was weaker than α-Syn119. However, truncated α-Syn133 has no effect on fibrillation of α-Syn.

**Figure 5:**
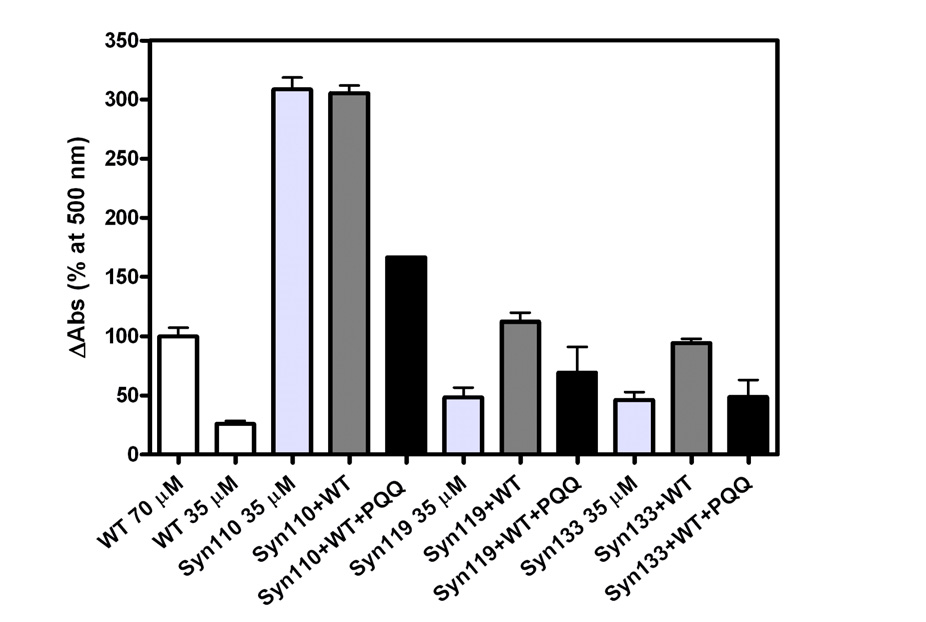
**Impact of C-terminal-truncated α-synuclein on the aggregation of full length of α-synuclein**. 70 μM full-length α-Syn, 35 μM truncated variants or a mixture of 35 μM full-length α-Syn and 35 μM truncated variants (Syn110+WT, Syn119+WT, Syn133+WT) were incubated in PBS buffer, pH 7.4, with stirring at 37°C in the absence (white bar or gray bar) and presence of 280 μM PQQ (black bar). The light scattering at 500 nm was used to monitor the total aggregation. n = 3 and error bar = standard deviation.

The light-scattering assay showed that the aggregation of full-length of α-Syn was increased 3-fold by mixing with α-Syn110. When α-Syn119 was mixed with full-length α-Syn, the OD_500 _was slightly increased (Fig. 5). But α-Syn133 did not enhance the aggregation of full-length α-Syn. It indicated that α-Syn110 and α-Syn119 enhanced the aggregation of full-length α-Syn, but not α-Syn133.

### PQQ prevents the fibril formation of truncated α-synuclein

**Figure 6:**
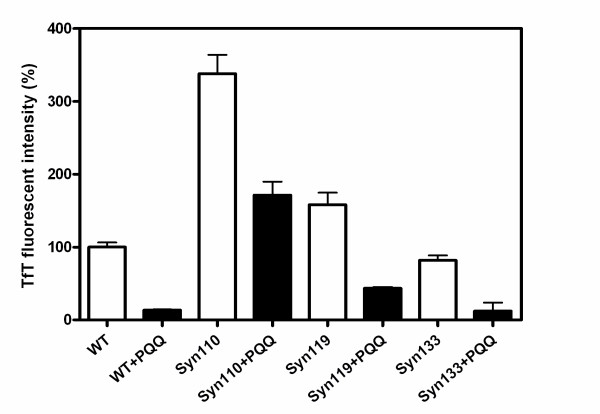
**Inhibition of the amyloid fibril formation of C-terminal-truncated α-synuclein by PQQ**. Full-length α-Syn (70 μM) and truncated variants (70 μM) were incubated in PBS buffer, pH 7.4 with stirring at 37°C in the absence (white bar) or presence of 280 μM PQQ (black bar). Fibril formation was measured by TfT fluorescence. n = 3 and error bar = standard deviation.

**Figure 7:**
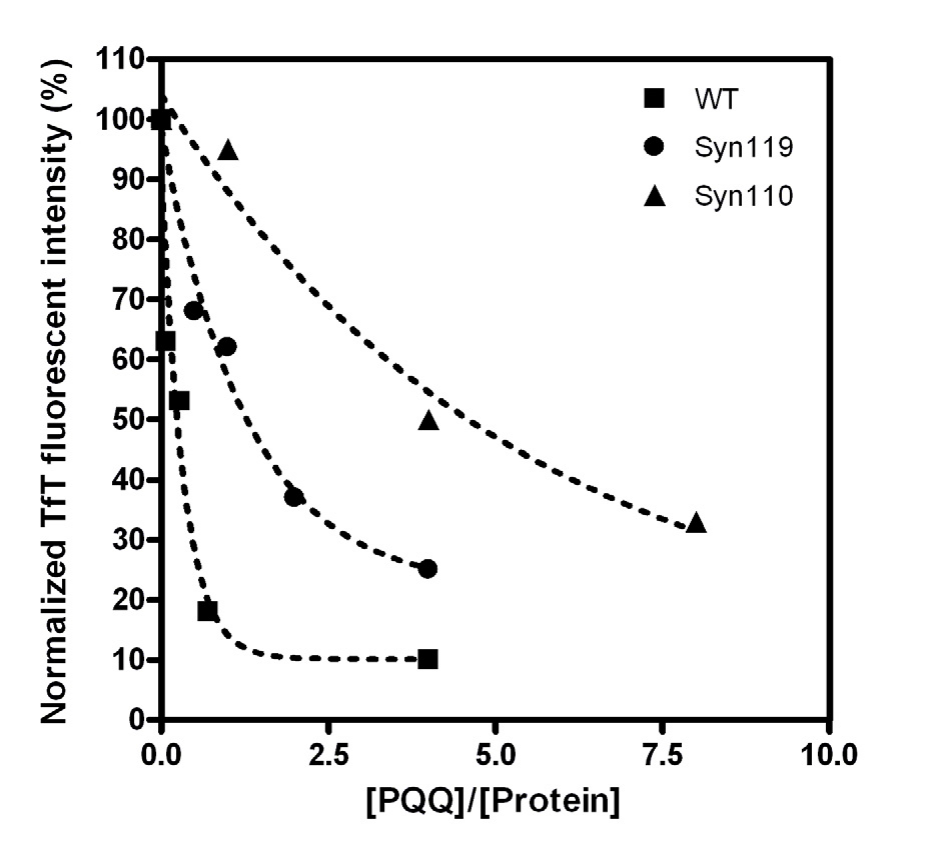
**The inhibitory effect of PQQ on the fibril formation of full-length or C-terminal-truncated α-synuclein at several concentrations**. Full-length α-Syn (white squares), truncated α-Syn119 (white circles) and truncated α-Syn110 (white triangles).

**Figure 8:**
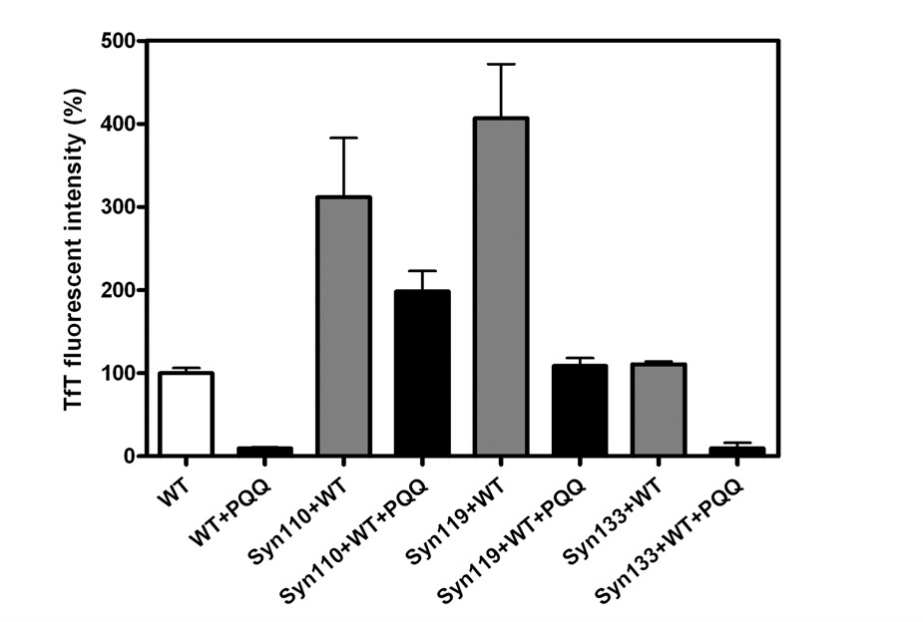
**Inhibition by PQQ of the amyloid fibril formation of C-terminal-truncated α-synuclein mixed with full-length α-synuclein**. 70 μM full-length α-Syn or a mixture of 35 μM full-length α-Syn and 35 μM truncated variants was incubated in PBS buffer, pH 7.4 with stirring at 37°C in the absence (white bar or gray bar) and in the presence of 280 μM PQQ (black bar). Fibril formation was measured by TfT fluorescence.

We have previously reported that PQQ prevents the aggregation and fibril formation of full-length α-Syn, and here we investigated whether or not PQQ also prevents the fibril formation of truncated α-Syn. It has been reported, in agreement with this study, that C-terminal truncation accelerates α-Syn fibril and oligomer formation. Also, truncated α-Syn promotes the fibril formation of full-length α-Syn. However, the presence of PQQ in truncated α-Syn solutions resulted in the dramatic prevention of fibril formation in our study (Fig. 6). Upon the addition of 280 μM PQQ, the fibrils formed in 70 μM truncated α-Syn110, α-Syn119, and α-Syn133 decreased to less than 50, 27, and 14.8% of the fibrils formed in the absence of PQQ, respectively. The higher the concentration of PQQ, the less truncated α-Syn fibrils were formed (Fig. 7). This indicates that the inhibitory effect is PQQ-dose-dependent. In addition, fibril formation in the mixture of truncated and full-length α-Syn was markedly inhibited (Fig. 8). In the presence of PQQ, moreover, the light scattering did not increase significantly, indicating that PQQ prevents the formation of truncated α-Syn aggregates ((Fig. 3) and (Fig. 5)). These results indicate that PQQ prevents the amyloid fibril formation and aggregation of truncated α-Syn as well as full-length α-Syn in a PQQ-concentration-dependent manner.

### PQQ decreases the cytotoxicity of truncated α-synuclein

**Figure 9:**
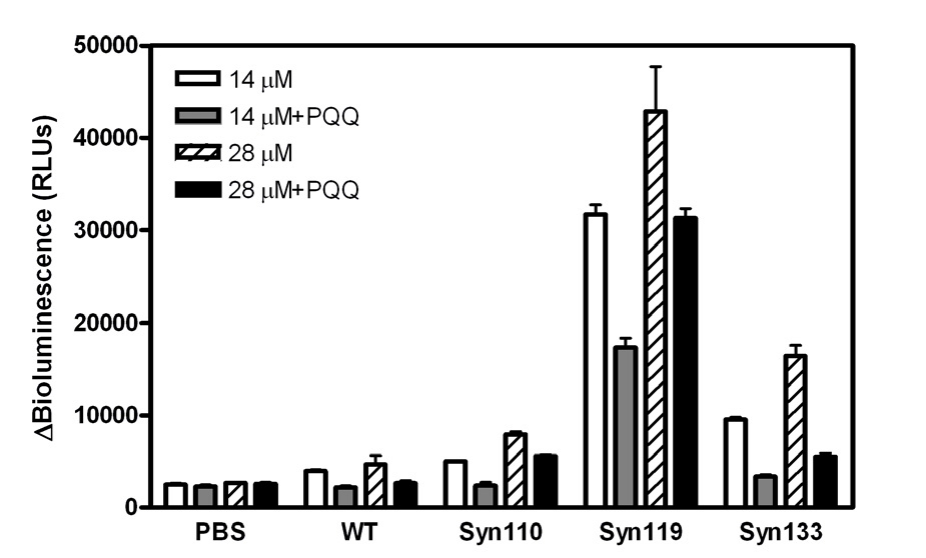
**C-terminal-truncated α-synuclein-mediated cytotoxicity can be mitigated by PQQ**. Full-length α-Syn and truncated α-Syn (final concentration 14 or 28 μM) were added to PC12 cells with shaking at 37°C in the absence or presence of 200 μM PQQ. After 96 h of incubation, the release of adenylate kinase from the damaged cells was measured by the luminescence of luciferase. n = 3 and error bar = standard deviation.

Previous studies have shown that aggregation and fibrillization of amyloid protein is toxic to neural cells [10,12,29,30]. We evaluated the effect of PQQ on the cytotoxicity of truncated variants to cultured PC12 cells. PC12 is a rat kidney cell line frequently used in the study of normally developing nervous systems. Full-length and truncated α-Syn samples were added to PC12 cells and incubated in the presence or absence of PQQ. These samples were then further cultured for four days with shaking at 37°C. We then evaluated the cytotoxicity of full-length and truncated α-Syn by adenylate kinase assay (AK assay), which measures quantitatively the release of AK from the damaged cells. The released AK converts ADP to ATP, which is measured by the bioluminescence assay, thus allowing the accurate and sensitive determination of cytotoxicity. Upon incubation of PC12 cells with 14 μM α-Syns (full-length αSyn, α-Syn110, α-Syn119, and α-Syn133), the bioluminescence increased to 1500, 3600, 28200, and 7300 RLUs, respectively. This indicates that the C-terminal-truncated α-Syn is highly toxic to PC12 cells, as opposed to the full-length α-Syn (Fig. 9). Addition of 28 μM α-Syn results in 1.4- to 1.8-fold higher bioluminescence than with 14 μM α-Syn, indicating that the cytotoxicity truncated α-Syn is dose-dependent.

**Figure 10:**
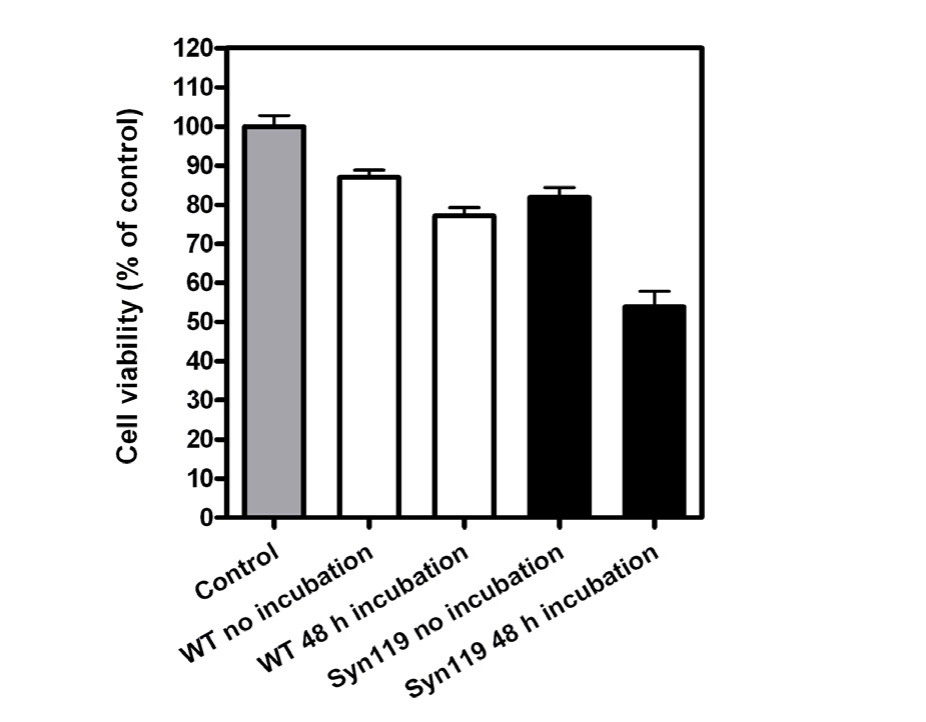
**C-terminal-truncation enhanced the cytotoxicity of α-synuclein**. Percentage of cell viability of PC12 cells added 21 μM of fresh or incubated α-Syn from *n *= 3 independent experiments. The results are expressed as a percentage of the value of "control" (PBS) (gray bar), which is set as 100%. Values are expressed as mean ± SD. Full-length α-Syn (white bar), and α-Syn119 (black bar).

The cytotoxicity evaluation results of α-Syn119 based on MTT assay are in good agreement with those from the adenylate kinase assay, as α-Syn119 is more toxic than wild-type, full-length α-Syn (Fig. 10). However, surprisingly, when PC12 cells to which truncated α-Syn was added were incubated with 200 μM PQQ, the bioluminescence decreased to 30-70% of that of the samples not treated with PQQ. The significant decrease in release of AK from damaged cells, suggests that the presence of PQQ greatly decreases the cytotoxicity of truncated α-Syn.

**Figure 11:**
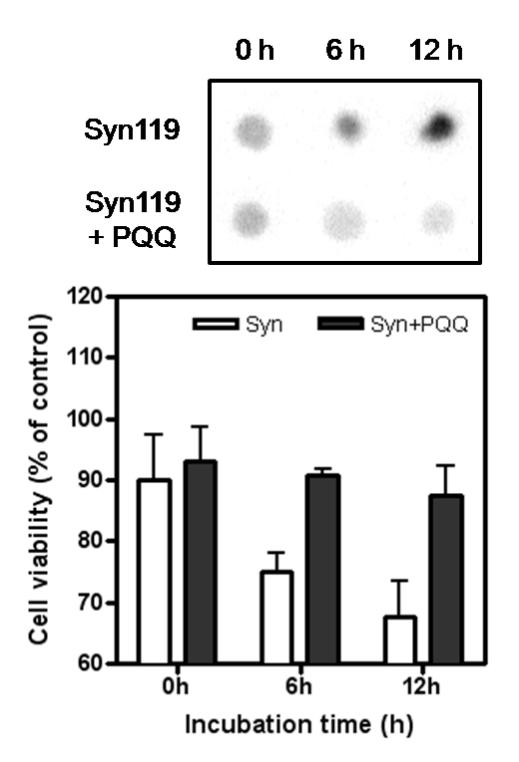
**Formation of C-terminal-truncated α-synuclein oligomer can be reduced by PQQ**. The dot blot analysis of truncated α-Syn119 sample with or without PQQ using anti-oligomer antibody, A11. 0 ~ 12 h incubated α-Syn119 was stained in membrane. Same samples were used to cell viability assay. 21 μM of incubated α-Syn119 in several time added to U2-OS cells. The results are expressed as a percentage of the value of "control" (PBS) (white bar), which is set as 100%. Values are expressed as mean ± SD, *n *= 3. Truncated α-Syn119 alone (white bar), and α-Syn119 + PQQ (black bar).

Recent studies have suggested that the oligomeric intermediate of fibrillation has a higher toxicity than the fibril. For the therapeutic application of PQQ, it is therefore important to investigate the effect of PQQ on both the oligomer formation and cytotoxicity of truncated α-Syn. We investigated the formation of oligomers of truncated synuclein119 with and without PQQ by dot blotting analysis using the anti-oligomer-specific antibody A11 [31]. We incubated 200 μM truncated synuclein119 in the absence or presence of a four-fold molar excess of PQQ and sampled at several times. In the absence of PQQ, the staining intensity of the α-Syn119 increased during the 12-hour time course (Fig. 11), reflecting the accumulation of oligomer. In contrast, the amount of oligomer did not change in the presence of PQQ. The cytotoxicity of these incubated samples, measured by adding them to U2-OS cells and culturing for 24 hours, increased in an oligomer dose-dependent manner. In the presence of PQQ, the cytotoxicity of a 12-hour incubated α-Syn119 was only 10%, compared to the 30% cytotoxicity of the α-Syn119 incubated 12 hours without PQQ. This clearly indicates that the cytotoxicity of α-Syn is related the oligomer formation by the truncated α-Syn119, and PQQ prevents the cytotoxicity of truncated α-Syn119 by inhibiting the formation of oligomers.

## Discussion

In this work, we prepared recombinant truncated α-Syn110, α-Syn119, and α-Syn133 and investigated the aggregation and fibril formation of these proteins *in vitro*. Also, we investigated the inhibitory effect of PQQ on the fibril formation and cytotoxicity of C-terminal-truncated α-Syn. It is the first attempt to characterize the aggregation and fibril formation of α-Syn119 and α-Syn133. It is possible that these truncated α-Syn variants play an important role in the pathogenesis of PD and related diseases, because C-terminal-truncated α-Syn119 and α-Syn133 have been found in both the normal and the pathogenic brain [18]. It is commonly understood that rapid aggregation and fibril formation occurs upon the truncation of the acidic C-terminal of α-Syn, which contains 10 glutamates and 5 aspartates. By truncation of negative charge acids, the repulsion between C-terminals of α-Syn was decreased and the possibility of interaction between hydrophobic regions contained NAC was increased. As a result, the aggregation and/or fibril formation were accelerated. In fact, α-Syn110 and α-Syn119 did show high aggregation and fibrillization speeds and rates compared to full-length α-Syn in our present study.

Unexpectedly the result of TfT fluorescence assay indicated that α-Syn133 slowed down the fibrillation in comparison with full-length α-Syn, whereas, α-Syn133 has almost equivalent aggregation with full-length α-Syn was observed. We understood that the speed of fibril formation of α-Syn133 decreased whereas the final quantity of fibril and aggregates are in the same level as those for wild type. Considering that the fibril elongation process is influenced by the status of soluble oligomers, the decreased kinetics in fibril formation of α-Syn133 might suggest the change of oligomeric status of α-Syn133.

Some studies have investigated the C-terminal-truncated α-Syn seeding mechanism, in which the truncated fragments nucleate full-length α-Syn aggregation through the formation of hybrid protofibrils and then fibrils [17,20,32,33]. This shortens the lag-time of fibril formation. When truncated α-Syn110 and α-Syn119, but not α-Syn133, coexisted with full-length α-Syn, it dramatically enhanced the total fibril formation of α-Syn. It indicated that co-existence of full length and truncated synuclein119 have synergistic effect on aggregation and fibrillation. Alzheimer's disease results mainly from the accumulation of amyloid β (1-42), though the total amount is lower than that of amyloid β (1-40). Likewise, it is presumed that the induction of the aggregation of full-length α-Syn by a small amount of C-terminal-truncated α-Syn is critical to its pathogenesis. Therefore, it is important to develop an inhibitor for the aggregation and fibrillization of truncated forms of α-Syn as well as full-length α-Syn.

PQQ exists in several plants and animals, and has been drawing attention as a novel nutritional factor due to its positive effect on cellular growth and its potential as a key pharmacological compound. Pharmacological research has addressed the issue of PQQ as a potential antioxidant and redox modulator. We have previously reported that PQQ prevents the fibril formation of full-length α-Syn and also influences the conformational change of α-Syn [28].

In our present study, PQQ significantly decreased the aggregation and fibril formation of truncated α-Syn as well as full-length α-Syn. Furthermore, in the presence of PQQ, aggregation and fibril formation in mixtures of C-terminal-truncated and full-length α-Syn were inhibited. This is the first report of an inhibitor for the aggregation and fibril formation of C-terminal-truncated α-Syn. In the presence of 4-fold molar PQQ, the fibril formation of full-length α-Syn and α-Syn133 was prevented to 10% of the fibrils formed in the absence of PQQ. However, in the case of α-Syn110 with 4-fold molar PQQ, fibril formation decreased only by 50%. The fibril formation of α-Syn119 decreased to 30% under the conditions of the experiment, as monitored by TfT fluorescence assay. This indicates that PQQ inhibits fibrillization in all of these C-terminal-truncated α-Syns, but that the potency of its inhibitory activity is different in each case (Fig. 7).

We have previously reported on the UV spectrum of α-Syn incubated with PQQ, which showed remarkable differences from the typical absorbance of PQQ or α-Syn alone. This suggests that PQQ forms a conjugate with α-Syn and that PQQ has reactive quinone groups, just as the oxidized form of baicalein [23]. Therefore, it is highly probable that PQQ binds with α-Syn via a Schiff base. Likewise, it is conceivable that PQQ binds to C-terminal-truncated α-Syn via a Lys residue. Although the number of Lys residues in C-terminal-truncated α-Syn is equal to that in full-length α-Syn, the inhibitory effect of PQQ is different between full-length α-Syn and truncated α-Syn. Based on the results of the TfT fluorescence assay, it is considered that the difference in the efficacy of PQQ's inhibitory activity derives from the speed of aggregation and fibrillization of each variant of truncated α-Syn. Since the aggregation and fibril formation of truncated α-Syn is faster than those of full-length α-Syn, in the case of truncated α-Syn, the reaction rate of binding with PQQ may be lower. As a result, until fibril formation begins, the instances of PQQ binding with truncated α-Syn will be fewer than with full-length α-Syn. We have reported that not only PQQ, but also PQQ-conjugated α-Syn lowered fibril formation [28]. This "feedback effect" gives PQQ considerable leverage in its inhibition of full-length α-Syn aggregation. In the case of C-terminal-truncated α-Syn, however, the effect is reduced by the ever decreasing quantity of PQQ-conjugated α-Syn.

It was known that amyloid proteins are toxic to culture cell and the expression of amyloid proteins was induced the damage of neurons in many neurodegeneration models. It is therefore important to evaluate the effect of PQQ on the cytotoxicity of amyloid proteins to develop an adequate therapy strategy. In this study, we measured the cytotoxicity of C-terminal-truncated α-Syn by adenylate kinase assay, MTT and ATP assay. The results show that C-terminal-truncated α-Syn was much more toxic to PC12 cells in a dose-dependent manner than full-length α-Syn. Especially the cytotoxicity of truncated α-Syn119 was distinctly different from that of other α-Syn variants. Under the experimental conditions, the luminescence intensity was 20-fold higher than that of full-length α-Syn. Interestingly truncated α-Syn133 is more toxic to PC12 cells than full-length α-Syn and α-Syn110. As we mentioned above, the decreased kinetics in fibril formation of α-Syn133 might suggest the change of oligomeric status of α-Syn133. In this respect, our interpretation that the increased toxicity of α-Syn133 owes to the change in the oligomeric status is in consistent with the experimental observation. We believe that truncation of the seven C-terminal residues affected the oligomeric status, consequently change the fibrillation kinetics.

Recently some studies suggested the intermediate of fibrillation, oligomer, has a high toxicity than fibril. It was known that some inhibitor of α-Syn aggregation increased the cytotoxicity of α-Syn despite the aggregation and fibril formation was significantly decreased [11]. These reports suggested that inhibitors induced the stable state of oligomer, intermediate of aggregation, and increased the cytotoxicity more. Other studies showed cytotoxicity of amyloid proteins was dependent on the conformations of oligomers as followed references [34,35], not the property of fibrillation. Thus, these studies supported that the fibrillation tendency of each variant dose not directly correlate their cytotoxicity effects. We showed that formed oligomer of truncated α-Syn119 was related to cytotoxicity of α-Syn and PQQ could inhibit the cytotoxicity of truncated synuclein119 by preventing the oligomer formation. In the progress of fibril formation, oligomerization is essential step for the fibrillation. From the results of kinetics of truncated synuclein in the presence of PQQ, the lag-time of fibrillation was delayed by PQQ which means that the formation of seeds for elongation of fibril, were prevented by PQQ. Together with results of cytotoxicity, it suggested that PQQ also could decrease the formation of truncated synuclein oligomers. This suggests that PQQ is a strong candidate for future anti-PD reagent compounds.

## Conclusions

Here, we showed that α-Syn110 and α-Syn119 has a high propensity of aggregation and enhanced the fibrillation of α-Syn. Although both of α-Syn119 and α-Syn133 showed a high cytotoxicity to culture cell, α-Syn133 showed a similar aggregation with full-length α-Syn and no acceleration effect. PQQ dramatically inhibits fibril formation in C-terminal-truncated α-Syn, as well as in mixtures of full-length α-Syn with truncated variants. Moreover, PQQ decreases the cytotoxicity of truncated α-Syn. Together with other findings on the inhibitors of amyloid proteins, this suggests that inhibitors other than PQQ, which also bind to target proteins, could prevent the aggregation and fibrillation of truncated α-Syn in a similar manner. In any case, PQQ is a strong candidate for a reagent compound in the treatment of PD and related diseases.

## Methods

### Chemicals

Thioflavin T (TfT) was purchased from Sigma. Pyrroloquinoline quinone was kindly donated by Mitsubishi Gas Chemical Company, Inc.

### Recombinant DNA technology

The full-length α-Syn structural gene was subcloned into the pET-28a(+) vector (Novagen). To construct the expression vectors of C-terminal-truncated α-Syn, pET-28a(+)-α-Syn vector was used to carry out site-directed mutagenesis using a QuikChange^® ^Site-Directed Mutagenesis Kit (Stratagene) with the following oligonucleotides inserted as the stop codon: 5'-GAAGGAGCCCCACAGGAATAAATTCTGGAAGATATGCCTGTG-3' (truncated α-Syn110), 5'-CTGGAAGATATGCCTGTGGATTAAGACAATGAGGCTTATGAAATGCCT-3' (truncated α-Syn119) and 5'-CCTTCTGAGGAAGGGTATTAAGACTACGAACCTGAA-3' (truncated α-Syn133). The sequence of the inserted gene fragment was confirmed by automated DNA sequencing (PerkinElmer ABI Model 310).

Expression vectors containing full-length α-Syn and truncated variants were thus constructed. The proteins used in this study were expressed in *Escherichia coli *BL21 (DE3). α-Syn expression was induced by the addition of 0.3 mM isopropyl-1-thio-β-D-galactopyranoside (IPTG). The cells were disrupted using a French press (Ohtake Works, Inc.), and the supernatant was collected by centrifugation (18,500 *g*, 10 min, 4°C). After boiling the collected supernatant at 100°C for 20 min, it was centrifuged (18,500 *g*, 10 min, 4°C) and dialyzed against buffer A (20 mM Tris-HCl, pH 8.0). After further centrifugation (12,000 *g*, 20 min, 4°C), the supernatant was loaded onto a Resource Q column (for full length α-Syn, truncated α-Syn119 and α-Syn133) or a Resource S column (for truncated α-Syn110) (GE Healthcare Bio-Science Corp.) and eluted with a 0-0.5 M NaCl gradient in buffer A. The eluted fractions were analyzed by SDS-PAGE, and the fractions containing α-Syn were dialyzed against PBS buffer (8.1 mM Na_2_HPO_4_, 1.4 mM KH_2_PO_4_, 137 mM NaCl and 2.7 mM KCl, pH 7.3). The concentrations of the proteins were determined by means of a DC protein assay kit (Bio-Rad).

### Preparation of aggregated solutions and TfT fluorescence & light-scattering assay

Purified α-Syn was concentrated to ca. 3.0 mg/ml using Amicon Ultra-15 filters (Millipore) in PBS buffer, pH 7.3, centrifuged (150,000 *g*, 1 h, 4°C) to remove any aggregates, and adjusted to 2.0 mg/ml. PQQ was dissolved in the same buffer at the defined concentration. The reaction solution contained 70 μM full-length or truncated α-Syn in the presence or absence of PQQ. Mixtures of full-length α-Syn and truncated α-Syn with or without 280 μM PQQ were also prepared. These samples were incubated on a 96-well plate in 200 μl of PBS buffer, pH 7.3, with 0.02% NaN_3 _at 37°C with shaking at 700 rpm. Fibril formation was monitored by thioflavin T (TfT) fluorescence. Aliquots of 5 μl were removed from the incubated samples and added to 250 μl of 25 μM TfT in PBS buffer, pH 7.3. TfT fluorescence was recorded at 486 nm with excitation at 450 nm using an ARVO MX 1420 multilabel counter (PerkinElmer). The lag time analysis of fibril formation was calculated from fitting-curve of ThT fluorescence was performed by PRISM (GraphPad Software). To compare the total aggregation of full-length and truncated α-Syn, the light scattering was studied. After incubation, 60-μM samples were measured at 500 nm. Usually, the formation of aggregations is monitored at 330 nm. However, PQQ has a typical absorbance at around 340 nm; so, the light scattering was monitored at 500 nm

### Cell culture and cytotoxicity analyses of truncated α-synuclein

PC12 cells (ATCC CRL1721) were routinely cultured in RPMI-1640 medium (Sigma) containing 5% fetal calf serum, 10% horse serum and 1% penicillin-streptomycin, and maintained at 37°C in a humidified incubator with 5% CO_2_/95% room air. For the cytotoxicity studies, the medium was removed and fresh medium was gently added before plating.

The cytotoxicity was measured by adenylate kinase assay. In brief, PC12 cells were plated at a density of 10,000 cells per well on CC3-coated 96-well plates (Nunc) in 100 μl of Opti-MEM medium. After 24 h of incubation, full-length or truncated α-Syn was added at the indicated concentration, and 200 μM PQQ was also added in half of the wells. The cells were incubated for an additional 4 days with shaking at 500 rpm, 37°C. The release of adenylate kinase from the damaged cells was measured using a ToxiLight^® ^Bioassay Kit obtained from LONZA [36,37]. Fifty μl of culture medium were removed from the each well and added to 100 μl of the reaction substrate. After incubation at room temperature for 5 min, the luminescence was measured on an ARVO MX 1420 multilabel counter (PerkinElmer). The bioluminescence of the control cells treated with PBS or PQQ instead α-Syn was also measured in the same way. PQQ had no effect on the AK assay as the luminescence with PBS was identical to that with PBS plus PQQ. The results shown in (Fig. 9) are after subtraction of these background values.

The cytotoxicity was measured by MTT and ATP assay. In brief, PC12 or U2-OS cells were plated at a density of 10000 cells per well on CC3-coated 96-well plates in 100 μl of fresh medium. After 24 h of incubation, full-length or truncated α-Syn119 was added at final concentration of 20 μM and cells were incubated for an additional 24 h. In MTT assay, cellular redox activity of tetrazolium formazan, 2-(2-methoxy-4-nitrophenyl)-3-(4-nitrophenyl)-5-(2,4-disulfophenyl)-2H tetrazolium were measured Cell Counting Kit-8 (DOJINDO) [38]. After replacement to fresh medium, 10 μl of stock WST-8 was added and the incubation was continued for another 2~3 h. Absorbance values at 450 nm were determined with an ARVO MX 1420 multilabel counter. The absorbance of the cells treated with PBS instead α-Syn was also measured in the same way as control (100%). In ATP assay, cell viability was measured by CellTiter-Glo Luminescent Cell Viability Assay Kit (Promega) following the manual. After replacement to 100 μl fresh medium, 100 μl of stock solution was added and the incubation was continued for 5 min. The luminescence values were determined with an ARVO MX 1420 multilabel counter. The luminescence of the cells treated with PBS instead α-Syn was also measured in the same way as control (100%).

### Dot blot analysis

Purified α-Syn119 was concentrated using Amicon Ultra-15 filters (Millipore) in PBS buffer, pH 7.3, centrifuged (150,000 *g*, 1 h, 4°C) to remove any aggregates, and adjusted to 3.0 mg/ml in the absence or presence of 800 μM PQQ. These samples were incubated in a 2 ml tube, in 1.2 ml of PBS buffer, pH 7.3, at 37°C with shaking at 700 rpm. The incubated α-Syn119 was sampled in several times. 1 μl of each sample applied to a nitrocellulose membrane, blocked with 10% skim milk in PBS containing 0.01% Tween 20 (TBS), at room temperature for 1 h, washed three times for 5 min each with TBS and incubated for 1 hr at room temperature with the anti-oligomer antibody A11 (0.1 μg/ml in TBS). The membranes were washed three times for 5 min each with TBS, incubated with anti-rabbit IgG diluted 1:10,000 in TBS and incubated for 1 hour at room temperature. The blots were washed three times with TBS and developed with ECL chemiluminescence kit from Amersham-Pharmacia (Piscataway, NJ).

## Competing interests

The authors declare that they have no competing interests.

## Authors' contributions

JK carried out all the studies, performed the acquisition of data and drafted the manuscript. HR, MK and NK participated in experimental designs and preparation of recombinant proteins. KS supervised and designed the whole project and contributed to the data interpretation. All authors read and approved the final manuscript.
